# Identification of a Peptide from *In vivo* Bacteriophage Display with Homology to EGFL6: A Candidate Tumor Vasculature Ligand in Breast Cancer

**DOI:** 10.4172/2155-9929.1000178

**Published:** 2014-04-01

**Authors:** Benjamin M Larimer, Susan L Deutscher

**Affiliations:** 1 Research Service, Harry S. Truman Veterans Memorial Hospital, Columbia, MO, USA; 2 Department of Biochemistry, University of Missouri, Columbia, MO, USA

**Keywords:** Phage display, Molecular imaging, Peptides, EGFL6, Breast cancer, Tumor vasculature, SPECT/CT

## Abstract

**Background:**

A crucial step in tumorigenesis is the recruitment of novel vasculature to the site of neoplasia. Currently, a number of high throughput techniques are employed to identify genes, mRNA and proteins that are aberrantly expressed in tumor vasculature. One drawback of such techniques is the lack of functional *in vivo* data that they provide. Bacteriophage (phage) display has been demonstrated *in vivo* to select peptides that home to tumors and tumor vasculature. The peptides can be compared to sequences of putative cancer-related proteins, in order to identify novel proteins essential for tumorigenesis.

**Objectives:**

It was hypothesized that an *in vivo* selection for phage which targeted human breast cancer xenografts could identify peptides with homology to cancer-related proteins for *in vivo* imaging of breast cancer.

**Methods:**

Following four rounds of *in vivo* selection in human MDA-MB-435 breast cancer xenografted mice, peptide 3-G03 was discovered with significant homology to a putative secreted protein termed EGFL6. Egfl6 mRNA is upregulated in several transcriptomic analyses of human cancer biopsies, and the protein may play a role in tumor vascularization.

**Results:**

Egfl6 mRNA expression was demonstrated in MDA-MB-435 cells and EGFL6 protein was secreted from these cells. Based on homology of 3-G03 to EGFL6, an EGFL6 peptide was synthesized and shown to target MDA-MB-435 cells. EGFL6 peptide was radiolabeled with ^111^In and analyzed for biodistribution and tumor imaging capabilities. Single photon emission computed tomography imaging revealed uptake of the peptide in a manner consistent with other tumor vasculature targeting agents.

## Introduction

Much effort has been placed on identifying the process of human tumor vascularization [1]. As tumor size increases, new vasculature is required to provide blood flow and nutrients to the growing malignancy, a process termed angiogenesis [2]. The network of blood vessels in each organ, including tumors, is differentiated by tissue specific expression of ligands and receptors [3]. These proteins serve as a molecular address, and in the case of tumorigenesis, may prove effective for delivery of imaging agents or cytotoxic drugs [4]. Although tumor vasculature proteins such as vascular endothelial growth factor, α_v_β_3_ integrin, and platelet derived growth factor are well known, resistance to therapies targeting these proteins reveals that tumor vasculature is a complex system that remains incompletely characterized [5]. The ability to identify and help validate novel ligands and their corresponding receptors responsible for tumor angiogenesis would prove advantageous. Not only could the ligands serve as potential targeting vectors for imaging applications, but blockade of receptors could prevent increased blood supply for a tumor and limit its growth.

In order to determine novel cancer biomarkers, techniques must be used that illuminate cancer-specific ligands and receptors. Deciphering the differential protein expression between malignant and non-malignant cells is often attempted using high throughput screening methods, due to the complex nature of the tumor microenvironment. One process for identifying novel cancer-specific ligands and receptors is mRNA profiling, which has been used to identify genes with significantly higher transcription levels in tumors [6–8]. Human cancer transcriptional profiles have served to create a reservoir of hypothetical tumor interacting transcripts, encoding for tumor vasculature proteins such as adlican, collagen type-XI alpha-1, glycoprotein M6B and epidermal growth factor-like domain multiple 6 (*egfl6*) [9]. The *egfl6* transcript, in particular, was first reported in several fetal tissues and human glioma tumor biopsy samples using a high throughput screening by hybridization technique [10]. Recently, several human tumor biopsy transcription analyses have indicated *egfl6* mRNA is expressed at high levels in meningioma, glioma and ovarian and breast carcinomas, while levels in normal tissues were virtually undetectable [6,8,11–13]. The reports of tumor specific *egfl6* expression suggest a need for investigation into its possible role in tumorigenesis.

The *egfl6* gene encodes for an approximately 60 kDa secreted protein with Epidermal Growth Factor (EGF) structural homology [14]. Although EGFL6 has been detected at the mRNA level in numerous cancers, the protein has not been detected in carcinoma cells and little is understood regarding its *in vivo* function. *In vitro*, full-length recombinant EGFL6 has been demonstrated to induce migration and angiogenesis in endothelial cells through activation of the extracellular signal-related kinase pathway [14]. These data suggest that EGFL6 may contribute to vascularization of new and perhaps malignant tissue. However, its roles in both development and tumorigenesis remain unclear.

One method of exploring the vast array of protein interactions and associations in a system such as the tumor vasculature is through bacteriophage (phage) display [15]. A single phage library can contain up to 10^9^ unique peptide sequences, offering a sizeable potential for selection of a peptide fragment of a natural ligand, such as EGFL6, which is thought to be specific to tumor vasculature [16]. Previous selections have demonstrated the feasibility of phage-based ligand identification, most notably isolating the peptide RGD [17]. The RGD motif was identified in 28 of 32 phage-displayed peptides selected for binding to α_5_β_1_ integrin, and consequently demonstrated to have high affinity for a number of integrins, including α_v_β_3_ [17,18]. Use of peptide phage display to identify binding epitopes, such as RGD, *in vitro* has spawned investigation into the ability of phage display to isolate tissue and tumor specific peptides *in vivo* [19]. *In vivo* phage display has previously identified tumor vasculature-homing peptides, and specific tripeptide sequences have been mapped to the vasculature of numerous human organs [20]. Additionally, our laboratory has developed a strategy for isolating not only tumor vasculature but also solid tumor-specific peptides [21]. These works indicate that phage can localize specifically to tumors, and recovered phage can be used to identify receptor-binding peptide epitopes.

It was hypothesized that *in vivo* phage display could be used to select peptides which mimic tumor-associated ligands. The peptides would serve not only as tumor imaging vectors but may also be used to help validate novel tumor biomarkers. Phage displayed peptides with homology to a known protein could help validate potential ligands identified by genomic or transcriptomic studies, or elucidate possible proteins underrepresented or absent from traditional proteomic analyses. To test this idea, a phage library was subjected to four rounds of *in vivo* selection in mice bearing human MDA-MB-435 breast cancer xenografts. Displayed peptides of phages recovered from the tumors were analyzed by the Basic Local Alignment Search Tool (BLAST). Although a number of peptides matched potential tumor related proteins, one peptide, with 9 of 14 amino acids identical (GTKSKCCYSLRRSS versus GTKLACCYGWRRNS) to EGFL6, was chosen for further study due to its significant homology and the growing evidence that EGFL6 is a potential tumor vasculature ligand. The tumor cell line used for selection, as well as several other cancer and non-cancer cell lines, was probed for mRNA and protein expression of EGFL6. Additionally, the tumor targeting and SPECT imaging properties of the EGFL6 peptide were investigated *in vivo*.

## Materials and Methods

### Materials

Materials for cell culture were purchased from Invitrogen (Carlsbad, CA). Unless otherwise specified, all reagents and materials were obtained from Sigma Chemical Co. (St. Louis, MO).

### Mouse strains and handling

Four- to 6-week-old Severe Combined Immunodeficient (SCID) out bred mice (Taconic, Germantown, NY) was maintained in approved pathogen-free institutional housing. Animal studies were conducted as outlined in the NIH Guidelines for the Care and Use of Laboratory Animals and the Policy and Procedures for Animal Research of the Harry S. Truman Veterans Memorial Hospital. MDA-MB-435 human breast cancer xenografts (5×10^6^) were established by subcutaneous injection into the flank of SCID mice. Mice were utilized when visual tumors (~1–3mm^3^) formed after approximately 5 weeks.

### Phage display selection and analysis

*In vivo* phage display was performed as described previously [21]. Briefly, a phage library containing approximately 1×10^12^ tetracycline transducing units of phage was injected into SCID mice bearing MDA-MB-435 human breast cancer xenografts. Phages were allowed to circulate for 1 h and following perfusion with Phosphate Buffered Saline (PBS), tumors were excised and frozen in liquid nitrogen. Tumors were manually homogenized and bound phages eluted by incubation with 2.5% (w:v) 3-[(3-cholamidopropyl) dimethylammonio]-1-propanesulfonate (CHAPS) solution. Recovered phages were used to infect log phase K91BK *E. coli* cells, amplified for 16 h in a 37°C incubator with shaking and purified by polyethylene glycol/sodium chloride precipitation [22]. Purified phages were quantified and used for subsequent rounds of selection, for a total of four selection rounds. Following the final round of selection, individual phages were isolated and their relevant DNA was sequenced in order to ascertain the displayed foreign peptide of each phage. Peptide sequences were then queried using the BLAST search program for sequence homology to proteins with known or putative cancer correlations [23].

### EGFL6 RT-PCR

In order to assay *egfl6* mRNA expression, three human breast cancer cell lines, MDA-MB-435, MDA-MB-468, SK-BR-3, and a normal cell line HEK-293, were grown to 80% confluency in T75 flasks and their RNA was isolated using Trizol (Ambion, Life Technologies, Grand Island, NY). Total RNA was quantified by spectrophotometry and 500 ng was reverse transcribed to cDNA using SuperScript III Reverse Transcriptase (200 units/µL) and oligodT primers (2.5 µM) (Life Technologies, Grand Island, NY). The subsequent cDNA was utilized for PCR reactions with *egfl6* specific primers previously demonstrated to amplify the gene of interest [10]. The primers [(5’-CGGGATCCCTGTGCTACGTCGCCCTGGAC-3’) and (5’-CGGAATTCACTGGCGCAGGCGGTGATCTCCTT-3’)] were diluted to 10 µM and added to 2 µL of cDNA for the reaction. The cycling conditions were 98°C for 30 s for one cycle, followed by 30 cycles of 98°C for 10 s, 60°C for 30 s and 72°C for 30 s. Primers for beta-actin were used as a loading control. PCR products were run on 1% agarose gels and visualized by ethidium bromide staining.

### Immunoassay

Each cell line was analyzed by immunoassay for EGFL6 protein expression in the cell lysate and supernatant using a polyclonal anti-EGFL6 antibody (Prestige Antibodies, Sigma, St. Louis, MO). Cells were grown to 80% confluency, supernatant harvested and both cells and supernatant were incubated with laemmli buffer (2% w:v sodium dodecyl sulfate, 10% glycerol, 60 mM Tris, 0.01% bromophenol blue). Protein concentrations were determined by Bio-Rad protein assay (Bio-Rad, Hercules, CA) and 500 µg of total protein was incubated with NuPAGE LDS sample buffer (Life Technologies, Grand Island, NY) at 80°C for 10 min and loaded onto NuPAGE Novex 4–12% Bis-Tris gels. Samples were electrophoresed for 90 min at 120 mV and transferred to 0.2 µm nitrocellulose membrane (Bio-Rad, Hercules, CA). Following transfer, membranes were blocked with 5% non-fat dry milk. Blocked membranes were incubated with anti-EGFL6 antibody diluted 1:50 in Tris buffered saline and 0.1% Tween-20 (0.1% TBST) for 10 min and vacuum aspirated by SNAP i.d. (Merck Millipore, Billerica, MA). Membranes were washed three times with 0.1% TBST and polyclonal anti-rabbit horseradish peroxidase-conjugated antibody diluted 1:1000 in 0.1% TBST was incubated with the membrane for 10 min. Vacuum aspiration and washing were completed as with the primary antibody. Membranes were developed using SuperSignal West Pico chemiluminescent substrate (ThermoFisher Scientific, Rockford, IL) and visualized using a VersaDoc Molecular Imager (Bio-Rad, Hercules, CA).

### Peptide synthesis

Synthesis of all peptides was accomplished with an Advanced Chem Tech 396 multiple peptide synthesizer using solid phase FMOC chemistry. Peptides were designed with an N-terminal GSG peptide spacer covalently linked at its N-terminus with either biotin or 1,4,7,10-tetraazacyclododecane-1,4,7,10-tetraacetic acid (DOTA) (Macrocyclic, Inc. Dallas, TX).

### Biotinylated peptide fluorescent microscopy

All cell lines were grown to 80% confluency and fixed with 4% paraformaldehyde. Cells were dried onto microscope slides overnight followed by rehydration with PBS. Slides were blocked with 5% BSA in PBS for 2 h at room temperature. Biotinylated EGFL6 peptide was diluted to 10 µM in PBS and 100 µL was added to each cell sample for incubation at room temperature for 1 h. Slides were washed 3 times with 0.1% TBST and cells were incubated with Neutravidin Texas Red (Life Technologies, Rockville, MD) diluted to 5 µg/mL in 0.1% TBST for 1 h at room temperature. Washing was performed in the same manner and cells were visualized by an epifluorescent Nikon T1-SM inverted microscope (Nikon, Melville, NY).

### DOTA-EGFL6 radiolabeling and purification

DOTA-EGFL6 was diluted to 1 mg/mL in water and incubated with 0.1 M ammonium acetate (pH 5) and 18.5 MBq of ^111^In at 80°C for 1 h. Reactions were quenched by addition of 10 µM EDTA. Radiolabeled peptide was purified by reverse phase HPLC using a linear gradient of acetonitrile plus 0.1% trifluoroacetic acid from 5–95% over 35 minutes. Acetonitrile was evaporated by nitrogen gas flow, and peptide was d iluted to appropriate concentration using sterile PBS.

### ^111^In-DOTA-EGFL6 cell binding

MDA-MB-435, MDA-MB-468, SK-BR-3 and HEK-293 cells were diluted to 2×10^6^ cells/mL in Dulbelco’s Modified Eagle Medium (DMEM) with 0.1 mg/mL BSA. ^111^In-DOTA-EGFL6 was diluted to 1×10^7^ CPM/mL in DMEM plus 0.1% BSA and 100 µL of peptide was added to 200 µL of cells and incubated at 37°C for 1 h. Cells were washed three times with ice-cold PBS with 1% BSA and counted via gamma counter.

### ^111^In-DOTA-EGFL6 Biodistribution and MicroSPECT/CT Imaging

DOTA-EGFL6 peptide was radiolabeled with ^111^In and purified peptide was prepared at 1.85 MBq/mL in sterile PBS. Three mice bearing MDA-MB-435 tumors were intravenously injected with ^111^In-DOTA-EGFL6 and sacrificed at 2 h. Organs and tissues pertinent to tumor uptake and clearance were excised, weighed, and counted by gamma counter. Percentage of injected dose per gram (%ID/g) of tissue was reported to normalize uptake by tissue mass.

Radiolabeled, purified ^111^In-DOTA-GSG-EGFL6 was diluted to 11.1 MBq in 100 µL of sterile PBS. The peptide was injected intravenously in a mouse bearing an MDA-MB-435 human breast cancer xenograft. Following injection, radiolabeled peptide was permitted to circulate for 2h, followed by euthanization by carbon dioxide. The treated mouse was imaged at the Harry S. Truman Veterans Memorial Hospital Biomolecular Imaging Center. Overnight (7h) image acquisition was performed with a Siemens Inveon Micro-SPECT/CT (Siemens, Knoxville, TN) equipped with mouse whole body 1.0 mm collimators. Data were processed with Inveon Research Workplace processing software and fan beam (Feldkamp) filtered back projection algorithms were used for reconstruction of the CT tomographic image.

## Results

### Phage display selection and analysis

**Figure 1:**
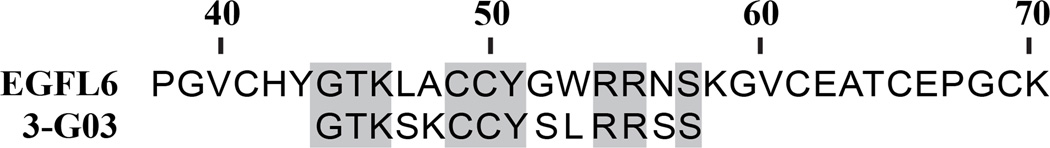
The *in vivo* selected peptide 3-G03 was aligned with EGFL6 for sequence comparison. A portion of the full length EGFL6 protein corresponding to the homologous selected peptide is displayed, with the hashed numbers representing the amino acid position from the N-terminus. Identical matches are highlighted

Completion of four rounds of *in vivo* phage display selection resulted in a sub-population of presumed human breast tumor-avid phage clones. DNA sequence corresponding to the foreign displayed peptide of 269 tumor avid phages was obtained and analyzed by the BLAST algorithm for homology to human cancer-related proteins [23]. For each peptide sequence, the top 10 matching proteins were evaluated for percent homology and previous identification as a cancer-related or putative cancer-related protein. One displayed peptide, from the phage clone 3-G03, revealed 64% identical homology to a protein termed EGFL6 (Figure 1). The match returned a score of 24.0 bits consisting of 9/14 positive identities and 0 gaps. The match returned an expected value of 4.9, indicating that random assignment of amino acids would only return approximately 5 random matches in the entire protein database. No other sequence returned a cancer-related protein and had sequence similarity greater than 50%.

### EGFL6 RT-PCR and immunoassay

**Figure 2:**
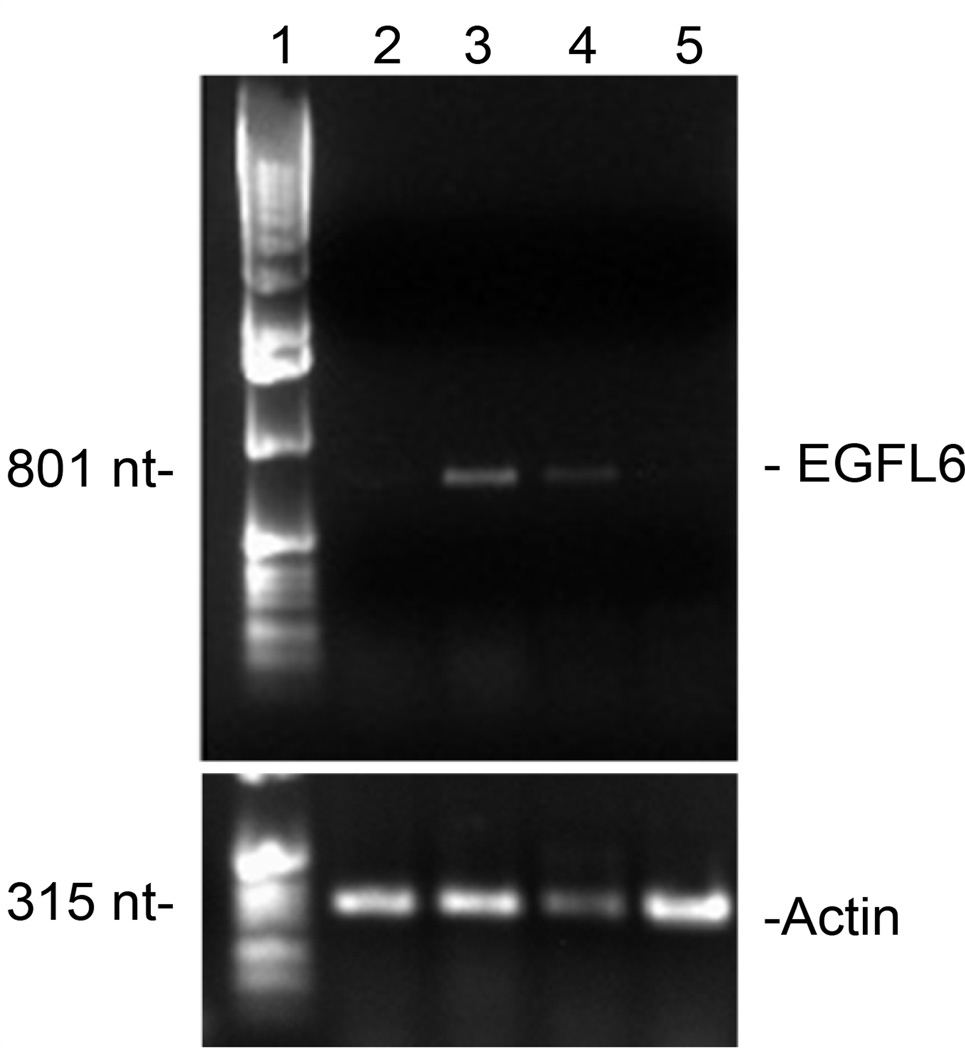
Isolated RNA from 3 human breast cancer cell lines and one human kidney cell line was reverse transcribed and amplified using EGFL6-specific primers. Bands of the expected nucleotide length were detected in MDA-MB-435 and MDA-MB-468 samples. Additionally, a primer for β-Actin was used as a loading control. Numbered lanes represent the following: 1) Ladder, 2) SK-BR-3, 3) MDA-MB-435, 4) MDA-MB-468, 5) HEK-293.

**Figure 3:**
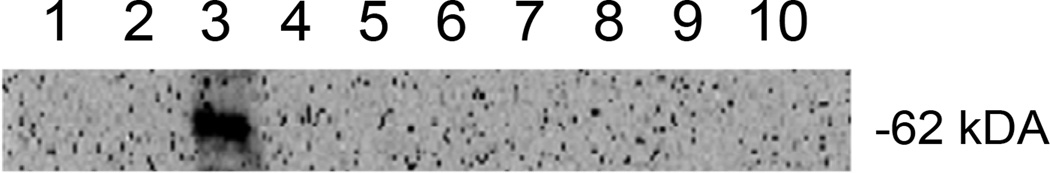
Total protein from cell lysates and cultured medium from corresponding cells were run on SDS-PAGE gels, transferred to nitrocellulose, and EGFL6 detected by immunoblot. One band was detected at the expected molecular weight for EGFL6 in the cultured supernatant of MDA-MB-435 cells. Numbered lanes represent the following: 1) Ladder, 2) MDA-MB-435 cell lysate, 3) MDA-MB-435 supernatant, 4) MDA-MB-468 cell lysate, 5) MDA-MB-468 supernatant, SK-BR-3 cell lysate, 6) SK-BR-3 supernatant, 7) HEK-293 cell lysate, 8) HEK-293 supernatant, 9) ladder, 10) loading buffer.

Due to the significant homology of the phage displayed peptide to EGFL6, it was inferred that the MDA-MB-435 cell line used for xenograft establishment for the *in vivo* phage display selection expressed EGFL6. In order to confirm *egfl6* transcription, RT-PCR was performed. Additionally, two more breast cancer cell lines, MDA-MB-468 and SK-BR-3, and a non-cancer cell line, HEK-293, were also assessed. PCR products from EGFL6 specific primers first used to identify the mRNA were visualized by ethidium bromide stain in agarose gel [10]. The results demonstrated a band corresponding to the expected fragment size of 801 nucleotides in the lane corresponding to MDA-MB-435 cDNA and a fainter band in the lane containing cDNA from MDA-MB-468 cells (Figure 2 and 3). No band was identified in the SK-BR-3 and HEK-293 lanes. Loading controls were accomplished using beta actin-specific primers producing a 315 nucleotide band, which was found in similar intensity in all cell lines analyzed.

Identification of *egfl6* at the mRNA level provided impetus to analyze protein expression of each cell line. Since EGFL6 is a secreted protein, both the cells and the cultured supernatant were used for immunoblotting. A band was identified in the supernatant of MDA-MB-435 cells that corresponded to the expected molecular weight of EGFL6, ~66 kDa. No band was detected in the supernatant of all other cell lines, nor the cell pellet of any cells examined, including MDA-MB-435.

### Biotinylated EGFL6 peptide fluorescent microscopy

**Figure 4:**
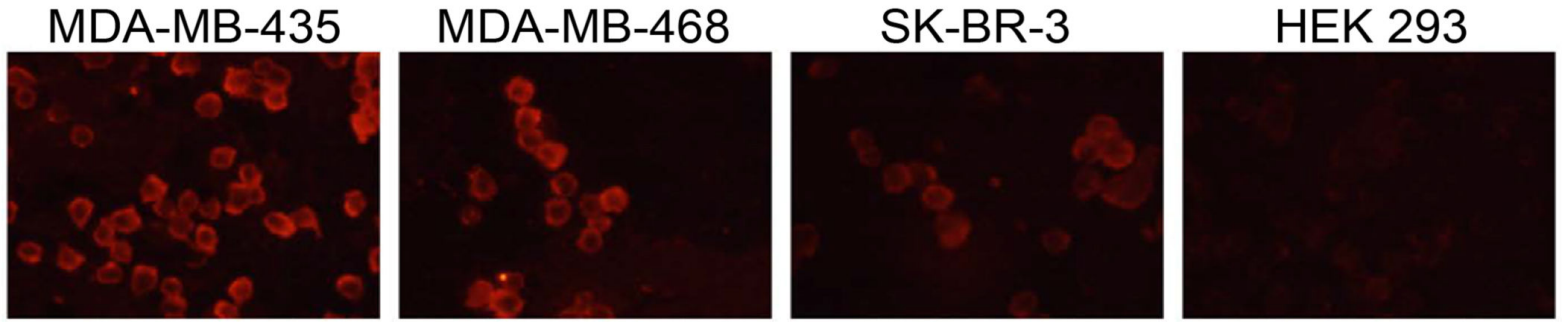
Human breast cancer and kidney cells were incubated with biotinylated peptides and visualized by fluorescent neutravidin. Fluorescent signal was detected in MDA-MB-435 and MDA-MB-468 cells, but not in SK-BR-3 or HEK-293 cells.

In order to confirm EGFL6 peptide affinity for MDA-MB-435 cells, the peptide sequence of EGFL6 (GTKWACCYGWRNSS) directly corresponding to the identified phage displayed peptide was synthesized and conjugated at the N-terminus with biotin for detection by fluorophore labeled streptavidin. Peptide binding was analyzed in the same four cell lines, MDA-MB-435, MDA-MB-468, SK-BR-3 and HEK-293, used for RT-PCR and immunoassay experiments. The results demonstrated the peptide bound strongly to MDA-MB-435 and MDA-MB-468 cells, while showing less binding to SK-BR-3 cells and no binding to HEK-293 cells (Figure 4).

### ^111^In-DOTA-EGFL6 cell binding

**Figure 5:**
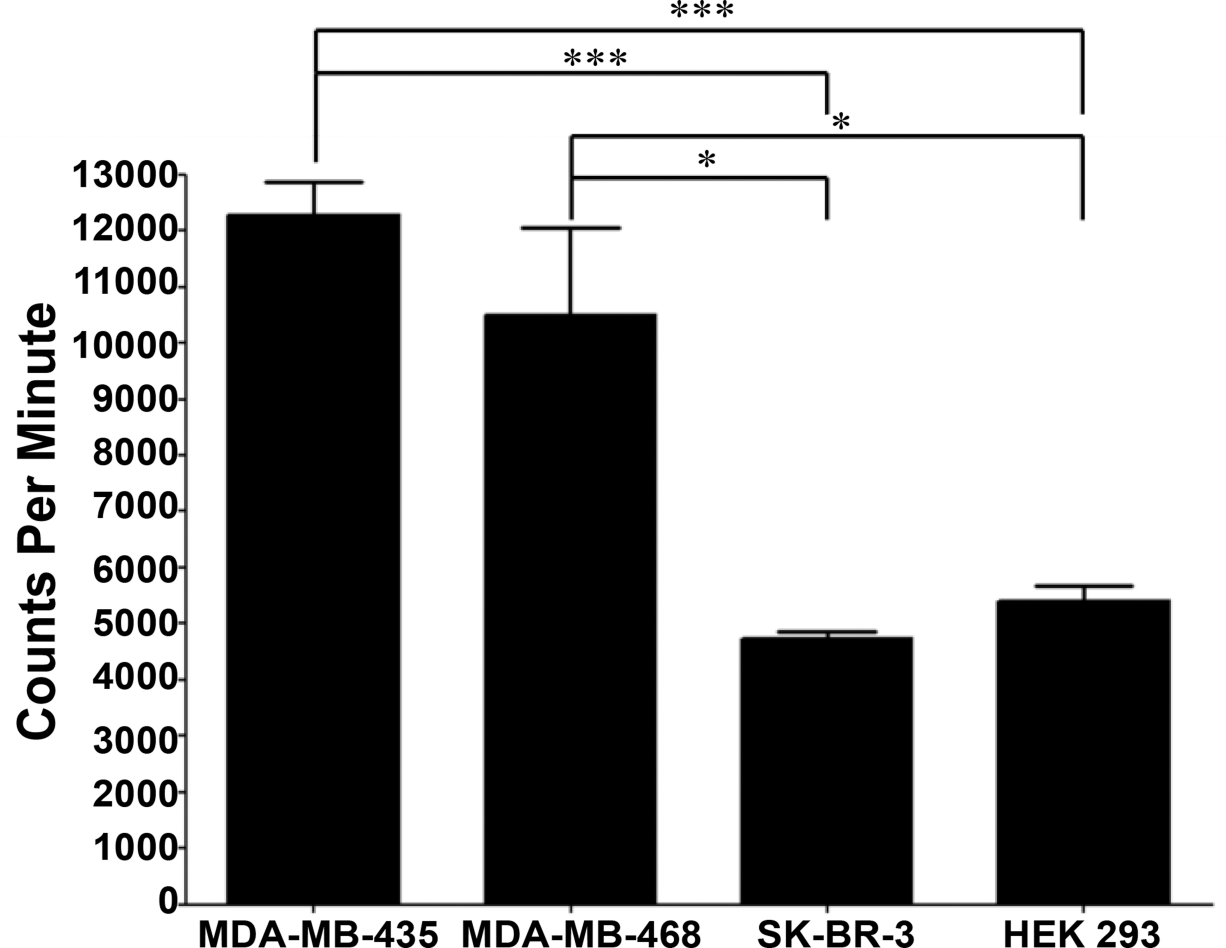
^111^In-DOTA-EGFL6 peptide was incubated with cell lines and binding was quantified. The peptide bound to MDA-MB-435 and MDA-MB-468 cells significantly higher than SK-BR-3 and HEK-293 cells. Error bars represent the standard deviation of 4 samples. *- *P* < 0.05 *** - *P* < 0.001.

After confirmation of cell binding with biotinylated peptide, the peptide was conjugated to the macrocyclic chelator DOTA for radiolabeled peptide binding assays. Radiolabeled peptide was examined for its ability to bind MDA-MB-435, MDA-MB-468, SK-BR-3 and HEK-293 cells. Peptide binding was determined to be 12.3 ± 1.0% (12284 CPM) of total peptide added for MDA-MB-435, 10.5% ± 2.7% (10508 CPM) for MDA-MB-468, 4.7 ± 0.2% (4731 CPM) for SK-BR-3 and 5.4 ± 0.5% (5411 CPM) for HEK-293 cells (Figure 5). The binding of ^111^In-DOTA-EGFL6 was significantly higher to MDA-MB-435 than SK-BR-3 (<0.001) and HEK-293 (*P* < 0.001) cells. Peptide binding was similar between MDA-MB-435 and MDA-468 cells and binding to MDA-MB-468 cells was also significantly higher (*P* < 0.05) than binding to SK-BR-3 or HEK-293 cells.

### ^111^In-DOTA-EGFL6 biodistribution

**Figure 6:**
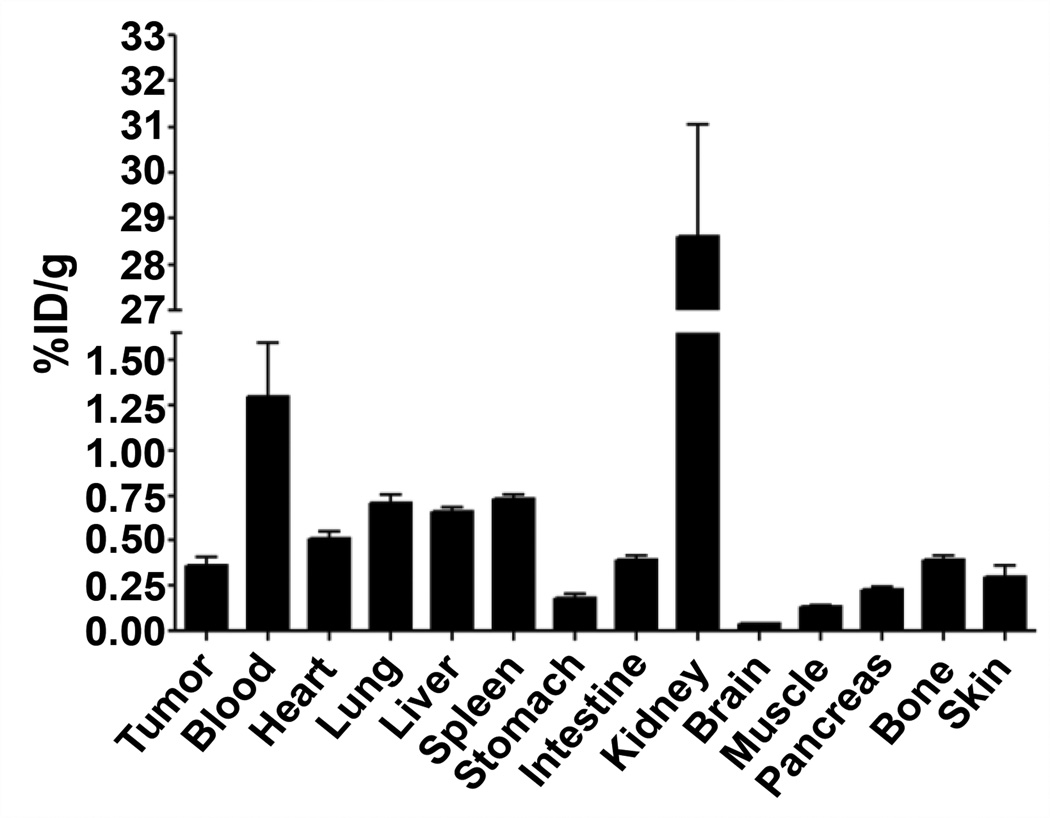
^111^In-DOTA-EGFL6 was injected into mice bearing MDA-MB-435 human breast cancer xenografts. Tissue biodistribution was analyzed 2 h post injection. Bars represent the mean of n=4 and error bars signify the standard deviation. %ID/g – percent injected dose/gram.

^111^In-DOTA-EGFL6 binding to MDA-MB-435 cells *in vitro* warranted *in vivo* analysis of the tumor targeting and non-target organ accumulation of EGFL6. In order to ascertain a preliminary understanding of the biodistribution, the pharmacokinetics of the peptide were analyzed at 2 h post-injection (Figure 6). Tumor uptake of the radiolabeled peptide was 0.36 ± 0.08 %ID/g, while blood retention of the peptide was 1.30 ± 0.51 %ID/g. Non-target organ accumulation was below 1.0 %ID/g for all organs measured, except for the kidneys. Kidney retention of the peptide was 28.61 ± 4.24 %ID/g. The tumor to blood ratio of the peptide was 0.27, while the tumor to muscle ratio was 2.7.

### ^111^In-DOTA-GSG-EGFL6 SPECT/CT imaging

**Figure 7:**
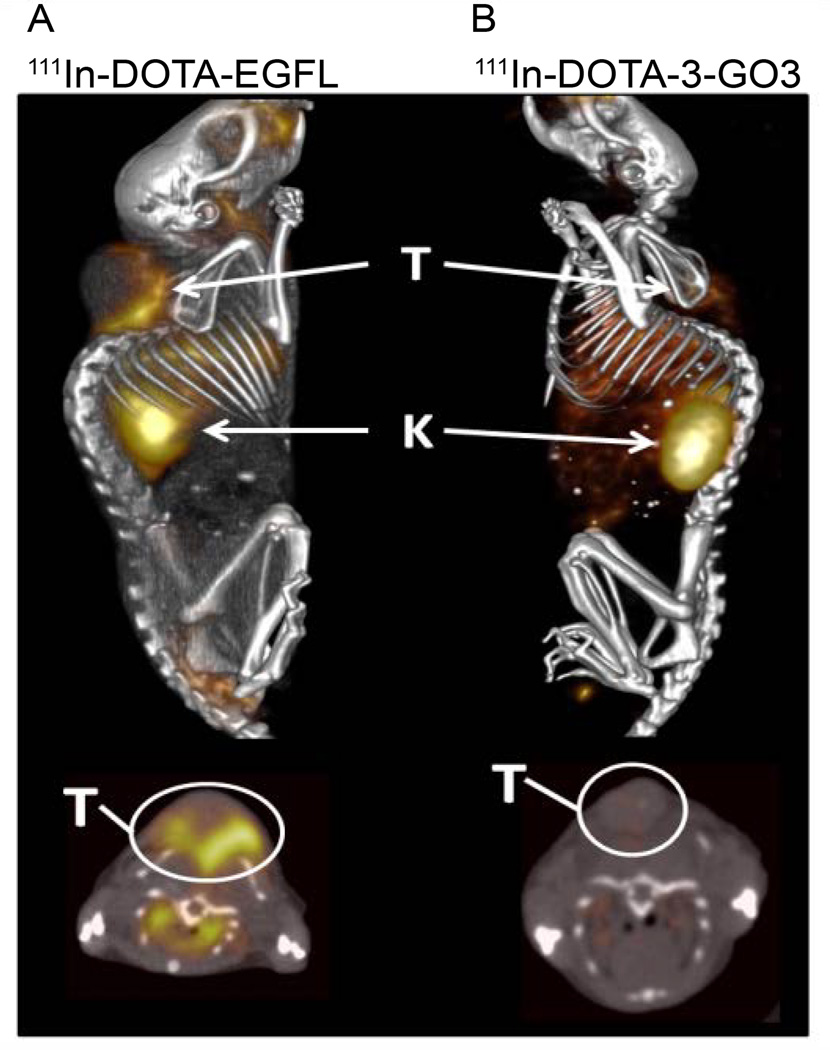
^111^In-DOTA-EGFL6 and ^111^In-DOTA-3-G03 were injected into MDA-MB-435 human breast carcinoma tumor bearing mice. SPECT/CT imaging was acquired 2 h post-injection. Uptake of ^111^In-DOTA-EGFL6 is consistent with tumor vasculature accumulation. Sequence specificity of the EGFL6 peptide was confirmed by using the 3-G03 peptide, which differs by only 5 amino acids. 3-G03 did not have a tumor uptake pattern similar to EGFL6, but did show similar kidney uptake, which is typical of radiolabeled peptides.

In addition to biodistribution, tumor imaging capabilities of ^111^In-DOTA-GSG-EGFL6 were explored. SPECT/CT images were collected at 2 h post-injection in order to correspond with the biodistribution data (Figure 7). Imaging revealed low tumor uptake, however, the interface between the xenograft and the muscle of the mouse had a strikingly high concentration of radiolabel. Kidney retention was also visible in the mouse, in addition to apparent cranio-facial, esophageal, and stomach intake, consistent with incidental ingestion of the radiolabeled peptide following oral cleaning of the injection site.

## Discussion

Angiogenesis in cancer is a key component of tumor progression and, as such, has received much attention in both basic and translational research. Clinically-approved drugs including bevacizumab (Avastin^®^; Genentech) and sunitinib (Sutent<; Sugen) in addition to counterparts in clinical trials, including VEGF-Trap_R1R2_ (Aflibercept; Regeneron Inc.) and vendatanib (Caprelsa; AstraZeneca), illustrate the major emphasis placed on preventing tumor angiogenesis [24–27]. Unfortunately, response to current anti-angiogenic therapy is generally transient, and relapse is common [28]. One hypothesis for the eventual ineffectiveness of anti-angiogenic therapy is circumvention of therapeutic blockade by upregulation of complementary growth factors and receptors, such as EGFR and fibroblast Bv8 [29,30]. Identification of the potential ligands that promote tumor angiogenesis is necessary to help overcome resistance to current therapies. Although the list of tumor-associated ligands is extensive, it is unlikely to be complete [1].

Proteins involved in tumorigenesis can be identified at three levels: the genome, transcriptome and proteome. While high throughput DNA, RNA and protein expression interrogation techniques provide unique advantages including large sample size, quantification and breadth of results, each by itself is incomplete. Genetic differences may not be transcribed, transcriptional differences may not be translated, and proteomic differences may be too small to be distinguished. Additionally, *ex vivo* analysis cannot directly investigate physiological location or function. In order to supplement these techniques, a method, such as *in vivo* phage display, can be used to explore protein interactions in a physiological environment. *In vivo* phage display has been utilized previously to map vascular signatures and identify novel tumor-associated proteins, such as proteome activator complex 28 and plectin-1 [20,31,32]. *In vivo* phage display provides two key supplementary features to current high throughput screening techniques: the ability to identify proteins that are expressed at low levels and not likely to be recognized by proteomic procedures, and the potential to identify targets that are biologically accessible for targeted imaging and therapy. Therefore, it was hypothesized that an *in vivo* phage display selection could identify tumor associated ligands by isolation of peptides with a similar sequence and function.

The *in vivo* selection enabled identification of peptides with tumor imaging capabilities in addition to providing an opportunity to explore whether peptides were similar to sequences of known or putative cancer proteins. Peptides corresponding to 267 phage clones recovered from human breast cancer xenografts were analyzed by the BLAST algorithm for homology to known cancer-related proteins. Since the sequences being analyzed were relatively short (15 amino acids), it was necessary to arbitrarily limit the scope of what was considered a match to greater than 50% homology. This greatly reduced the number of artificial hits that would be generated by random chance. Of the 267 peptides analyzed, only one, 3-G03, fit the criteria specified. 3-G03 (GTKSKCCYSLRRSS) matched a secreted protein, EGFL6 (GTKLACCYGWRRNS), with 64% homology and returned a score of 24 bits, primarily due to the number of identical matches (9) and the absence of gaps needed to make the identical pairs. It was interesting to note that the 9 identical pairs consisted of 2 tripeptide sequences, and an additional 4 peptide sequence with one mismatch. This is consistent with previous selections that have identified three amino acid sequences capable of mediating interaction between a ligand and a receptor, as is the case of RGD and several integrins [17]. Furthermore, tripeptide motifs have been demonstrated to target phage to specific organs *in vivo*, including the tumor vasculature [20]. The high homology between 3-G03 and EGFL6, in addition to the potential tumor vasculature promoting properties of the protein, led to further investigation into EGFL6 expression and peptide characterization.

Because a peptide with homology to EGFL6 was identified by the *in vivo* selection, it was assumed that the xenografted breast cancer cell line expressed EGFL6. For confirmation, RT-PCR was used to analyze *egfl6* transcription in three breast cancer cell lines and a control cell line. The results confirmed that *egfl6* was expressed in MDA-MB-435 cells, in addition to MDA-MB-468 cells, although at apparently lower levels. This result was consistent with identification of *egfl6* expression in biopsy samples from ovarian and breast cancer, as well as meningioma [6,8,13].

While *eglf6* mRNA has been identified, EGFL6 protein has not been confirmed in cultured carcinomas. In order to verify the *egfl6* transcript was translated, an immunoassay probing EGFL6 expression was performed. EGFL6 is a secreted protein; therefore immunoassay was performed on both the cultured supernatant and cell pellet of all cell lines previously analyzed. Interestingly, EGFL6 protein was only identified in the supernatant of MDA-MB-435 cells and not in MDA-MB-468 cells. Lack of detectable protein in MDA-MB-468 cells could have resulted from the protein concentration being undetectable by immunoassay, which is feasible due to the apparent diminished amount of mRNA detected by RT-PCR. Also EGFL6 translation in MDA-MB-468 cells may have been disrupted, as protein production in cancer cells has been demonstrated to be aberrant [33,34]. Additionally, the protein may be degraded rapidly under *in vitro* conditions by the cells. Detection of EGFL6 protein expression in cultured MDA-MB-435 cells confirmed the assumption that EGFL6 was expressed by the cell line used in the *in vivo* selection and provided motivation to further investigate the properties of the EGFL6 peptide.

Although EGFL6 protein expression was demonstrated by immunoblot, the affinity and specificity of the corresponding EGFL6 peptide for human breast carcinomas needed to be assessed. The exact peptide sequence for EGFL6 corresponding to the phage displayed homologue was selected for analysis. This approach was chosen to maximize the possibility that the properties of the peptide were mediated by the natural EGFL6 sequence, and not selected by phage display. EGFL6 peptide was chemically synthesized with a covalently linked biotin to determine *in vitro* peptide specificity for the cell lines previously analyzed. Fluorescent microscopy revealed strong binding to MDA-MB-435 and MDA-MB-468 human breast cancer cells. Binding was undetectable to SK-BR-3, a third human breast cancer cell line, and HEK-293 human kidney cells. Peptide affinity for the carcinomas corresponded with cell lines that expressed *egfl6* mRNA, even though the protein expression would suggest binding in only MDA-MB-435 cells. The receptor for EGFL6 is not known; therefore it is impossible to predict how the peptide or protein may bind to cells. A correlation between peptide binding and *egfl6* mRNA expression suggests that peptide uptake may predict cellular expression of the *egfl6* transcript. Obviously a short peptide sequence cannot fully mimic a full-length protein, which may contain several domains, post-translational modification sites and tertiary structure [10,14].

Identification of a peptide that bound to human breast carcinoma cells expressing EGFL6 offered the potential that the EGFL6 peptide could be used *in vivo* to detect tumorigenesis in xenografts expressing the potential tumor vasculature ligand. To evaluate the biodistribution and imaging properties of the EGFL6 peptide, it was necessary to conjugate the peptide to a radiometal chelator. Bifunctional macrocyclic DOTA was chosen due to its stable chelation with a number of radiometals, including indium-111, and well understood *in vivo* properties [35–37]. Prior to *in vivo* studies, retained specificity and affinity of the radiolabeled peptide was monitored by *in vitro* cell binding. ^111^In-DOTA-EGFL6 bound to MDA-MB-435 and MDA-MB-468 cells significantly higher than SK-BR-3 or HEK-293 cells, confirming retention of the breast cancer targeting properties of the peptide.

Radiolabeled, purified ^111^In-DOTA-EGFL6 was injected into mice bearing MDA-MB-435 human breast cancer xenografts for biodistribution studies. Surprisingly, tumor uptake was low at 0.36 ± 0.08 %ID/g. The tumor to blood ratio was also suboptimal at 0.27, but a tumor to muscle ratio of 2.7 indicated specificity for the tumor. A corresponding SPECT/CT image was acquired for further insight into the low tumor uptake. Image analysis correlated with the results of the biodistribution. Although the solid tumor lacked significant uptake of ^111^In-DOTA-EGFL6, there appeared to be a high concentration at the interface of the tumor and the site of xenograft formation, presumably a sight of high neovascularization [38]. The SPECT/CT image was consistent with other vasculature-targeted imaging agents, such as radiolabeled RGD peptide [39]. Absence of solid tumor-retention could be the result of peptide binding to its target at the novel vasculature and not permitting further diffusion into the tumor [40]. Another reason for low tumor uptake might be differential expression of the receptor for EGFL6 *in vitro* and *in vivo. In vivo* expression of the receptor may be limited to cells near the site of neovascularization, thus limiting uptake to the interface of tumor and normal endothelial cells [40]. Previous work had demonstrated the phage selected peptide, 3-G03, bound to MDA-MB-435 cells *in vitro*, and its sequence similarity to EGFL6 made ^111^In-DOTA-3-G03 a unique control to determine whether the precise EGFL6 sequence was necessary for its *in vivo* imaging properties. While a very slight tumor uptake was observed for ^111^In-DOTA-3-G03, the distinct vasculature-like binding observed with ^111^In-DOTA-EGFL6 was not present in the ^111^In-DOTA-3-G03 images. Kidney uptake, which is not sequence specific and instead a result of filtration of low molecular weight peptides, was present for both peptides. SPECT/CT imaging revealed that EGFL6 was targeted to the tumor-epithelial interface of human breast carcinoma xenografts, and the binding was specific to the EGFL6 peptide sequence.

## Conclusion

An *in vivo* phage display selection resulted in the identification of a potential tumor vasculature ligand, EGFL6. Additionally, a peptide with homology to EGFL6 was radiolabeled with ^111^In and used to image tumor vasculature by SPECT/CT. This data presents evidence that EGFL6 should be further investigated for its roles in tumorigenesis and as a possible imaging agent.

## Abbreviations

Egfl6: Epidermal Like Growth Factor-6
EGF: Epidermal Growth Factor
SCID: Severe Combined Immunodeficiency
Chaps: 3-[(3-Cholamidopropyl) Dimethylammonio]-1-Propanesulfonate
TBST: Tris Buffered Saline and Tween
DOTA: 1,4,7,10-Tetraazacyclododecane-1,4,7,10-Tetraacetic Acid
